# Complex samples logistic regression analysis of predictors of the current use of modern contraceptive among married or in-union women in Sierra Leone: Insight from the 2013 demographic and health survey

**DOI:** 10.1371/journal.pone.0231630

**Published:** 2020-04-16

**Authors:** Pascal Agbadi, Tagoe Twumwaa Eunice, Agyemang F. Akosua, Seth Owusu

**Affiliations:** 1 Department of Nursing, Faculty of Allied Health Sciences, College of Health Sciences, Kwame Nkrumah University of Science and Technology, Kumasi, Ghana; 2 School of Development Management, Ghana Christian University College, Kumasi, Ghana; 3 Department of Social Work, University of Ghana, Accra, Ghana; 4 African Institute for Mathematical Sciences, Adisadel-Cape Coast, Ghana; FHI360, UNITED STATES

## Abstract

**Background:**

Non-use of modern contraceptives among married or in-union women aged 15 to 49 years is a demographic and public health challenge. Studies on the predictors of contraceptive use among women in Sierra Leone are few, more than two decades old, and not nationally representative. This study aims to fill this gap by estimating the prevalence and the predictors of the current use of modern contraceptives among married or in-union women in Sierra Leone.

**Methods:**

This is a population-based study that used the 2013 Sierra Leone Demographic and Health Survey (SDHS) dataset. We performed complex samples logistic regression with 9,111 reproductive-age women. The analysis was done using the IBM SPSS Statistics version 21, and statistical significance was pegged at p≤0.01 and p≤0.05. The outcome of interest is the current use of modern contraceptives among reproductive-age married or in-union women in Sierra Leone, measured as ‘Yes’ (currently using a modern method) and ‘No’ (using a folkloric method, traditional method, and no method).

**Results:**

About 18.1% of reproductive-age women were currently using a modern contraceptive. The study found the following sociodemographic factors as positive correlates: being within the age group of 20–24 years [AOR = 1.52, CI: 1.05, 2.19], 25–29 years [AOR = 1.57, CI: 1.10, 2.19], 30–34 years [AOR = 2.31, CI: 1.59, 3.36], 35–39 years [AOR = 1.89, CI: 1.33, 2.70], 40–44 years [AOR = 1.68, CI: 1.12, 2.52], obtaining either a primary [AOR = 1.40, CI: 1.14, 1.71] or secondary level education [AOR = 1.34, CI: 1.02, 1.74], belonging to the category of women that condemned wife beating under only one condition [AOR = 1.37, CI: 1.03, 1.78], under two conditions [AOR = 1.45, CI: 1.08, 1.93], under three conditions [AOR = 1.73, CI: 1.28, 2.35], under four conditions [AOR = 1.91, CI: 1.34, 2.72], and under five conditions [AOR = 1.41, CI: 1.07, 1.85], having the ability to refuse sex [AOR = 1.46, CI: 1.23, 1.76], ever heard family planning on the radio [AOR = 1.30, CI: 1.08, 1.58], being sexually active four weeks prior to the survey [AOR = 3.90, CI: 3.14, 4.84], ever taken an HIV test [AOR = 1.67, CI: 1.39, 2.02], ever visited a health facility in the last 12 months [AOR = 1.73, CI: 1.44, 2.09], dwelling in a richer household [AOR = 1.32, CI: 1.01, 1.72], and dwelling in an urban area [AOR = 1.44, CI: 1.14, 1.81]. Exposure to family planning through print media (newspaper/magazine) was negatively associated with current use of modern contraceptive [AOR = 0.60, CI: 0.37, 0.96].

**Conclusion:**

The study provided in-depth insight into the sociodemographic predictors of the current use of modern contraceptives among married or in-union women in Sierra Leone. The study underscored the need to promote the protective factors of the current use of modern contraceptives and address the risk factors of the low prevalence of modern contraceptive use through policies, programs, and interventions in Sierra Leone.

## Background

Non-use of modern contraceptives among married or in-union women aged 15 to 49 years is a demographic and public health challenge. The global modern contraceptive prevalence (MCP) among this group is 56% [1]. The MCP among women living in high-income countries is 60%, which is twice as high as those living in low-income countries (29%) [1]. In Sierra Leone, MCP among married or in-union reproductive-age women is as low as between 15% and 20% [2,3]. Non-use of modern contraceptive (N-UMC) is associated with unplanned pregnancies that many times lead to unsafe abortions as well as a significant predictor of maternal and child morbidity and mortality [4–7].

Knowing that cost-effective public health programs and interventions hold the key to increasing the MCP and reducing the consequences associated with N-UMC, many countries and organizations have pledged their support as reflected in the sustainable development goal and the family planning 2020 initiative (FP2020). The sustainable development goals (SDG) 3.7 and 5.6 enjoins all signatory countries to advocate for universal access to sexual and reproductive health-care services, including access to family planning, information and education, and the integration of reproductive health into national strategies and programs [8]. The FP2020 is an initiative by numerous governments, private sector stakeholders and organizations in the world [9]. The goal of this initiative is to support the efforts of countries to increase the number of women and girls using contraceptives by 120 million by the end of 2020 by encouraging them to work together with civil societies, multilateral organizations, and donors [9]. Similar to the SDGs on contraceptive use, the FP2020 planned to achieve its aim through holistic policy design and implementation, adequate financial allocation and utilization, quality service delivery and the alleviating of socio-cultural barriers [9].

Many economic, cultural, and psychosocial factors act as a barrier to the use of modern contraceptive among women. Some of these factors include lack of formal education, fear of side-effects from the use of modern contraceptives, rural residence, cultural and religious prohibitions, poverty, and unavailability, limited access, and non-demand for family planning services [10–19]. Studies on the predictors of contraceptive use among women in Sierra Leone are few, more than two decades old, and not nationally representative [10,11]. These studies found that lower age at marriage, lower socioeconomic status, nonattainment of formal education, and Islamic religious affiliation are risk factors of low contraceptive usage [10,11]. To design programs and interventions that aim at increasing the prevalence of modern contraceptive use among women, it is crucial to have current studies that delineate factors that explain modern contraceptive use in Sierra Leone. This study fills this gap by estimating the prevalence of current use of modern contraceptives (CUMC) and examining the socio-economic and demographic predictors of CUMC.

## Methods and data

### Study design

This is a population-based study that used the 2013 Sierra Leone Demographic and Health Survey (SDHS) dataset to identify predictors of the current use of modern contraceptive (CUMC) among married and in-union women in Sierra Leone.

### Sample design and data collection

The sampling was done in two stages. First, primary sampling units (PSUs) were selected from the 2004 Sierra Leone Population and Housing Census (2004 SLPHC) sample frame [2]. In all, 435 PSUs (277 rural and 158 urban) were selected for the survey [2]. Thirty households were systematically selected from each PSU, and all women age 15–49 who were usual household members or who spent the night before the survey in the selected households were eligible for individual interviews [2]. The number of eligible 15–49 years old women interviewed in 2013 was 16,658. Data were collected by the use of questionnaires covering socioeconomic, demographic, and health indicators [2]. The sampling techniques and procedures that governed the SDHS are comprehensively reported elsewhere [2].

### Study and analytic sample

We restricted the analysis to women who were currently in-union. Consequently, women who were currently not in any amorous union, who never had sex, who were currently pregnant, and were infecund were excluded from the dataset [see Table 1]. The study sample selected was 8,942. We accounted for the multistage sampling design during analysis, which resulted in weighting the dataset using a weight variable in the dataset for reproductive-age women. Thus, the weighted study sample used for analysis was 9,111 reproductive-aged married or in-union women in Sierra Leone [see Table 1].

**Table 1:** Analytic sample selection process.

			Study sample
	Total cases available in the dataset		16,658
Excluded	Infecund women	473	
			**16,185**
Excluded	Women who never had sex	1,466	
			**14,719**
Excluded	Currently pregnant women	1,426	
This sample comprises of women who were: never in a union, currently in-union, and formerly in union			**13,296**
**Selected**	Currently in union/living with a man (subpopulation)		**9, 184**
Excluded	Observations with missing data on study variables	242	
Unweighted	Subpopulation with no missing data		**8,942**
**Weighted**	**Subpopulation with no missing data**		**9,111**

### Measures

#### Outcome variable

The outcome of interest is the current use of modern contraceptives among reproductive-age married or in-union women in Sierra Leone. Women were asked whether they were currently using any contraceptive method at the time of the survey. In this study, the responses [‘no method’ or ‘folkloric method’ or ‘traditional’ or ‘modern method’] to the contraceptive use question were dichotomized and measured as ‘Yes’ (currently using a modern method) and ‘No’ (using a folkloric method, traditional method, and no method).

#### Predictor variables

To explain the variability in women’s CUMC, the following fifteen variables were selected based on literature and their relevance [10–19]: the age, education, religion, attitude towards wife-beating, ability to refuse sex, ability to ask a partner to use a condom, exposure to family planning messages through the radio, newspaper, and the TV, recent sexual activity, ever been tested for HIV, visited health facility in last 12 months, household wealth, place of residence, and region of residence.

Apart from age, education, recent sexual activity, ever been tested for HIV, visited a health facility in last 12 months, household wealth, exposure to family planning messages through the radio, newspaper, and the TV, place of residence, and region of residence variables that were not re-coded, the categories of these variables were re-coded as follows: religion [‘Christian’, ‘Islam’, ‘others’ (Bahai, traditional, other, none)]; ability to refuse sex [‘No’ (No, Don’t know/not sure/depends)], ‘Yes’], ability to ask a partner to use a condom [‘No’ (No, Don’t know/not sure/depends)], ‘Yes’]. The attitude towards wife-beating was measured in the SDHS by asking the women to agree (yes = 0) or disagree (no = 1) to these five statements: (1) beating justified if the wife goes out without telling husband, (2) beating justified if wife neglects the children, (3) beating justified if wife argues with husband, (4) beating justified if the wife refuses to have sex with husband, and (5) beating justified if wife burns the food. The values of the responses were added to generate a composite score for condemning attitude towards wife-beating variable with the highest score being ‘5’ (in total disagreement that wife-beating is justified under all the five circumstances) and the lowest being ‘0’ (in total agreement that wife-beating is justified under all the five circumstances). The newly created condemning attitude towards the wife-beating variable was treated and interpreted as an ordered categorical variable.

### Statistical analyses

Statistical analyses were performed using the IBM SPSS Statistics version 21. The statistical significance thresholds for all analyses of the association between the criterion and the explanatory variables were pegged at the 1% and 5% level of significance (p≤0.01 and p≤0.05).

We used the following variables to account for the multistage sample design inherent in the DHS dataset during the descriptive, test of association, and logistic regression analyses: women's (individual) sample weight, sample strata for sampling errors, and cluster number. Given that we selected a subpopulation (women who were currently married/living with a man), we used the “Currently/formerly/never in union” variable to specify the subpopulation during the analysis. The DHS calculated the individual sample weights to six decimal places but are presented in the dataset without points. Thus, the sample weight for each case was computed by dividing the available weight in the dataset by 1,000,000 before using it in the analyses. This was done according to the guideline of DHS on handling the individual weight variable, which can be found on page 1.32 here [20].

#### Descriptive analyses

Sample characteristics were expressed as frequencies and percentages, and the chi-square test of independence was used to assess the association between CUMC and the predictor variables.

#### Regression analysis

Complex samples logistic regression was used to perform a multivariable analysis of predictors of CUMC.

### Ethical considerations

The 2013 SDHS protocol was reviewed and approved by the Sierra Leone National Ethics Committee and the Institutional Review Board of ICF International. Informed consent was also obtained from participants before they were interviewed. The SDHS is publicly available upon a simple, registration-access request, so no further ethical clearance was sought.

## Results

### Summary statistics of study variables

In the dataset, 1,646 (18.1%) reproductive-age women were currently using a modern contraceptive. A total of 6,574 (72.2%) women had no formal education. The majority of the women were Muslims (81.8%), had ever taken an HIV test (58.1%), dwelling in rural areas (72.5%), and residing in the northern region (39.6%). Summary statistics for all study variables are shown in Table 2.

**Table 2:** Summary statistics of study variables.

Study Variables	n* (%)
**CUMC**	
No	7465 (81.9)
Yes	1646 (18.1)
**Age**	
15–19	529 (5.8)
20–24	1286 (14.1)
25–29	1952 (21.4)
30–34	1697 (18.6)
35–39	1709 (18.8)
40–44	1015 (11.1)
45–49	923 (10.1)
**Education**	
No education	6574 (72.2)
Primary	1181 (13.0)
Secondary	1194 (13.1)
Higher	162 (1.8)
**Religion**	
Christian	1602 (17.6)
Islam	7457 (81.8)
Others	52 (0.6)
**Condemning attitude towards wife-beating**	
0	1482 (16.3)
1	979 (10.7)
2	1795 (19.7)
3	1251 (13.7)
4	656 (7.2)
5	2948 (32.4)
**Can refuse sex**	
No	2819 (30.9)
Yes	6292 (69.1)
**Can ask a partner to use a condom**	
No	6039 (66.3)
Yes	3072 (33.7)
**Heard Family Planning on Radio**	
No	4479 (49.2)
Yes	4632 (50.8)
**Read Family Planning on Newspaper**	
No	8841 (97.0)
Yes	270 (3.0)
**Heard Family Planning on TV**	
No	8514 (93.5)
Yes	596 (6.5)
**Recent sexual activity**	
Active last 4 weeks	5181 (56.9)
Not active last 4 weeks	3929 (43.1)
**Ever been tested for HIV**	
No	3815 (41.9)
Yes	5295 (58.1)
**Visited health facility in the last 12 months**	
No	6611 (72.6)
Yes	2500 (27.4)
**Household wealth**	
Poorest	1925 (21.1)
Poorer	1918 (21.0)
Middle	1923 (21.1)
Richer	1748 (19.2)
Richest	1597 (17.5)
**Place of residence**	
Urban	2508 (27.5)
Rural	6603 (72.5)
**Region of residence**	
Eastern	2176 (23.9)
Northern	3606 (39.6)
Southern	2021 (22.2)
Western	1308 (14.4)

*Rounded to a whole number

### Chi-square test of independence between the outcome and descriptive variables

A Chi-square test of independence was performed to ascertain the relationship between the CUMC and descriptive variables. The results indicated that women’s current use of modern contraceptives was statistically significantly associated with each study's descriptive variable. Details of the Chi-square test results are reported in Table 3.

**Table 3:** Chi-square test of independence between the outcome and predictor variables.

Study Variables	CUMC	
	No	Yes
	n* (%)	n* (%)
**Age**		
15–19	479 (90.5)	50 (9.5)
20–24	1076 (83.7)	209 (16.3)
25–29	1609 (82.4)	344 (17.6)
30–34	1298 (76.5)	399 (23.5)
35–39	1366 (79.9)	343 (20.1)
40–44	827 (81.5)	188 (18.5)
45–49	810 (87.8)	113 (12.2)
χ^2^ = 87.89; p-value = 0.000		
**Education**		
No education	5566 (84.7)	1008 (15.3)
Primary	920 (77.9)	261 (22.1)
Secondary	876 (73.4)	318 (26.6)
Higher	102 (63.0)	60 (37.0)
χ^2^ = 140.81; p-value = 0.000		
**Religion**		
Christian	1243 (77.6)	358 (22.4)
Islam	6180 (82.9)	1277 (17.1)
Others	42 (79.2)	11 (20.8)
χ^2^ = 24.23; p-value = 0.001		
**Condemning attitude towards wife-beating**		
0	1323 (89.3)	159 (10.7)
1	832 (85.1)	146 (14.9)
2	1486 (82.6)	309 (17.2)
3	967 (77.3)	284 (22.7)
4	504 (76.8)	152 (20.2)
5	2352 (78.8)	596 (18.1)
χ^2^ = 98.26; p-value = 0.000		
**Can refuse sex**		
No	2475 (87.8)	343 (12.2)
Yes	4989 (79.3)	1303 (20.7)
χ^2^ = 93.78; p-value = 0.000		
**Can ask a partner to use a condom**		
No	5080 (84.1)	958 (15.9)
Yes	2384 (77.6)	688 (22.4)
χ^2^ = 57.42; p-value = 0.000		
**Heard Family Planning on Radio**		
No	3882 (86.7)	596 (13.3)
Yes	3582 (77.3)	1050 (22.7)
χ^2^ = 131.91; p-value = 0.000		
**Read Family Planning on Newspaper**		
No	7261 (82.1)	1581 (17.9)
Yes	204 (75.6)	66 (24.4)
χ^2^ = 7.25; p-value = 0.045		
**Heard Family Planning on TV**		
No	7058 (82.9)	1457 (17.1)
Yes	407 (68.2)	190 (31.8)
χ^2^ = 79.67; p-value = 0.000		
**Recent sexual activity**		
Active last 4 weeks	3865 (74.6)	1317 (8.4)
Not active last 4 weeks	3600 (91.6)	329 (8.4)
χ^2^ = 429.76; p-value = 0.000		
**Ever been tested for HIV**		
No	3346 (87.7)	470 (12.3)
Yes	4119 (77.8)	1177 (22.2)
χ^2^ = 144.35; p-value = 0.000		
**Visited health facility in the last 12 months**		
No	5579 (84.4)	1031 (15.6)
Yes	1885 (75.4)	615 (24.6)
χ^2^ = 97.17; p-value = 0.000		
**Household wealth**		
Poorest	1658 (86.1)	267 (13.9)
Poorer	1655 (86.3)	263 (13.7)
Middle	1658 (86.2)	266 (13.8)
Richer	1360 (77.8)	388 (22.2)
Richest	1134 (71.0)	463 (29.0)
χ^2^ = 215.59; p-value = 0.000		
**Place of residence**		
Urban	1812 (72.2)	696 (27.8)
Rural	5653 (85.6)	950 (14.4)
χ^2^ = 215.24; p-value = 0.000		
**Region of residence**		
Eastern	1766 (81.1)	411 (18.9)
Northern	3118 (86.5)	488 (13.5)
Southern	1635 (80.9)	386 (19.1)
Western	946 (72.3)	362 (27.7)
χ^2^ = 131.04; p-value = 0.000		

*Rounded to a whole number

### Predictors of CUMC

The predictors in the complex samples multiple logistic regression model explained about 13.1% of the variability in the outcome variable [McFadden R^2^: 0.131]. Sociodemographic factors that positively associated with CUMC among the women were: being within the age group of 20–44 years, obtaining either a primary or secondary level education, having more than a zero point the five-point condemning attitude towards wife-beating scale, having the ability to refuse sex, ever heard family planning on radio, being sexually active four weeks before the survey, ever taken an HIV test, ever visited a health facility in the last 12 months, and dwelling in an urban area. Exposure to family planning through print media (newspaper/magazine) was negatively associated with CUMC. Details of the odds ratio with its corresponding 95% confidence intervals are reported in Table 4.

**Table 4:** Complex samples logit model of predictors of CUMC.

	OR [95% CI]	Std. Error
(Intercept)	0.01 [0.01, 0.02]**	0.26
**Age**		
15–19	1.0 [reference]	
20–24	1.52 [1.05, 2.19]**	0.19
25–29	1.57 [1.10, 2.19]**	0.18
30–34	2.31 [1.59, 3.36]**	0.19
35–39	1.89 [1.33, 2.70]**	0.18
40–44	1.68 [1.12, 2.52]**	0.21
45–49	1.01 [0.65, 1.57]	0.23
**Education**		
No education	1.0 [reference]	
Primary	1.40 [1.14, 1.71]**	0.10
Secondary	1.34 [1.02, 1.74]**	0.14
Higher	1.47 [0.93, 2.30]	0.23
**Religion**		
Christian	1.0 [reference]	
Islam	0.89 [0.74, 1.06]	0.09
Others	1.28 [0.46, 3.56]	0.52
**Condemning attitude towards wife-beating**		
0	1.0 [reference]	
1	1.37 [1.03, 1.78]**	0.14
2	1.45 [1.08, 1.93]**	0.15
3	1.73 [1.28, 2.35]**	0.16
4	1.91 [1.34, 2.72]**	0.18
5	1.41 [1.07, 1.85]*	0.14
**Can refuse sex**		
No	1.0 [reference]	
Yes	1.46 [1.23, 1.76]**	0.09
**Can ask a partner to use a condom**		
No	1.0 [reference]	
Yes	0.99 [0.81, 1.21]	0.10
**Heard Family Planning on Radio**		
No	1.0 [reference]	
Yes	1.30 [1.08, 1.58]**	0.10
**Read Family Planning on Newspaper**		
No	1.0 [reference]	
Yes	0.60 [0.37, 0.96]**	0.24
**Heard Family Planning on TV**		
No	1.0 [reference]	
Yes	1.20 [0.83, 1.74]	0.19
**Recent sexual activity**		
Not active last 4 weeks	1.0 [reference]	
Active last 4 weeks	3.90 [3.14, 4.84]**	0.11
**Ever been tested for HIV**		
No	1.0 [reference]	
Yes	1.67 [1.39, 2.02]**	0.10
**Visited health facility in the last 12 months**		
No	1.0 [reference]	
Yes	1.73 [1.44, 2.09]**	0.10
**Household wealth**		
Poorest	1.0 [reference]	
Poorer	0.98 [0.78, 1.23]	0.12
Middle	0.89 [0.70, 1.14]	0.12
Richer	1.32 [1.01, 1.72]*	0.14
Richest	1.16 [0.81, 1.67]	0.18
**Place of residence**		
Rural	1.0 [reference]	
Urban	1.44 [1.14, 1.81]**	0.12
**Region of residence**		
Eastern	1.0 [reference]	
Northern	0.92 [0.72, 1.18]	0.13
Southern	1.20 [0.94, 1.52]	0.12
Western	0.99 [0.72, 1.36]	0.16
Population size	9,111	
Strata	27	
Primary Sampling Unit	435	
McFadden R^2^	0.131	

** p≤0.01

* p≤0.05

## Discussion

Our study revealed that 20–44 years old women were more likely to use a modern contraceptive method compared to 15–19 years old women. This result concurs with the results of previous studies undertaken in Sierra Leone [10,11] and those of others done elsewhere [21–23]. These studies found that Papua New Guinean [21]. Burkina Faso [23], and Bangladesh [22] women’s use of a modern form of contraceptive increases with age. The young women may be at risk of low patronage of modern contraceptives because they may be less empowered compared to adult women to exercise their decision power to use a contraceptive. Also, these young women may find it difficult to access reproductive health services and resources that give them autonomy over their sexual life because the cultural norms in most parts of the country are not in support of modern contraceptive usage.

A number of women empowerment proxies in our study, such as education, condemning attitude towards wife-beating, and the ability to refuse sex, had a statistically significant association with CUMC. The results of our study revealed that obtaining at least a primary education, condemning the beating of wives, and having the ability to refuse sex facilitated the CUMC among married or in-union women. The protective effect of education and empowerment on modern contraceptive usage are facts in literature [13,14], and these explain why the strengthening of empowering pathways are included in the SDG 3.7 and 5.6 and the objectives of the FP2020 initiatives [8,9].

Religion was not statistically significantly related to CUMC in our study. However, in previous similar studies from Sierra Leone, religion was found to be a significant predictor with Muslim women being at risk of non-use of modern contraceptives compared to Christian [10,11]. Our finding suggests that the differences that exist between Christians and Muslims in terms of the current use of modern contraceptives may be losing its significance over time, but this needs further investigation.

We investigated the possibility of exposure to family planning through the media as a correlate of the CUMC. Our study revealed that hearing family planning messages through the radio was positively associated with CUMC. Sierra Leonean women's exposure to family planning messages through the TV was not a significant predictor of CUMC, and we unexpectedly found that women's exposure to family planning through the newspaper was negatively associated with CUMC. The radio is an effective public health medium for altering unhealthy behaviors and attitudes and to encourage people to adopt healthy lifestyles. Similar to our result, the positive effect of exposure to radio on public health behavior has been observed in other studies [24,25]. These studies found that women who listen to the radio/TV or frequently exposed to any form of mass media were more likely to adopt preventive measures that eventually translate into improved health outcomes [24,25].

Women who were sexually active four weeks before the survey were more likely to have used a modern form of contraceptive. This is encouraging because of the benefits that come with using a modern form of contraceptive. Except for women who want to bear a child, using a modern form of contraceptive prevents unwanted pregnancies, unsafe abortions and improves health outcomes [26]. This result is similar to the results of another study. The study found that women who were sexually active, knowing the consequences associated with unplanned pregnancies and sexually transmitted diseases, were more likely to use a modern form of contraceptive [27].

Our study revealed that women who have ever taken an HIV test and have ever visited a health facility in the last 12 months were positively associated with CUMC. These results are expected, for HIV tests occur mostly in a healthcare setting, making HIV testing a plausible proxy measure for access to healthcare. As revealed by our study that ever visiting the hospital is a predictive factor for CUMC, it appears that women who have had access to healthcare facilities were educated and admonished to place a premium on the use of modern contraceptives. In the hospitals and other healthcare facility tiers, there are public health spots at the outpatient departments (OPD) that educate patients on several preventive measures to adopt for a healthy lifestyle.

Our study found that women from urban areas were more likely to use modern contraceptives compared with women in rural settings. This finding could be explained by a couple of factors. Compared to rural dwellers, urban dwellers often have adequate knowledge of modern contraceptives and are much more involved in decisions related to their reproductive health [28]. Also, women living in rural areas might be less educated and less exposed to media campaigns and messages on modern contraceptives; as a result, they may be less informed to decide on modern contraceptive uptake [24].

Our study revealed that the region a woman resided in Sierra Leone has no significant relationship with the current use of modern contraceptives. The results of our study contradict multiple studies from other countries that have shown that the region of residence is statistically significantly related to CUMC [29–31].

Our study revealed that women from richer households were more likely to use modern contraceptives compared to the poorest households. Household wealth generally is a predictive factor of many health outcomes, including the use of modern contraceptives as found by other studies [24,32–34]. Household wealth may have provided Sierra Leone women with an opportunity for exposure to health information as well as giving them the purchasing power to afford a modern form of contraceptives.

### Study limitation and strength

The results of this study are based on a cross-sectional survey; therefore, the analyses cannot establish causal relationships. Thus, the conclusions in this study are restricted to associations between the predictors and the outcome variable. Another potential limitation is the possibility of reporting bias on the part of women in answering the survey question on contraceptive use. Additionally, our use of secondary data for analysis has limited us to choose from available variables in the dataset. Besides the limitation, an observed strength of the study is the use of the SDHS dataset that is nationally representative and large, which makes it possible for nationwide generalization.

## Conclusion

This study sought to investigate the predictive factors of the CUMC among married and in-union women in Sierra Leone. The study found the following sociodemographic factors as positive correlates: being within the age group of 20–44 years, obtaining either a primary or secondary level education, condemning wife-beating, having the ability to refuse sex, ever heard family planning on the radio, being sexually active four weeks before the survey, ever taken an HIV test, ever visited a health facility in the last 12 months and dwelling in an urban area. The study underscored the need to promote the protective factors of the current use of modern contraceptives and address the risk factors of the low prevalence of modern contraceptive usage through policies, programs, and interventions.

## Supporting information

S1 Dataset The minimal dataset underlying the results of the study. (SAV)
